# Pharmaco-epidemiological research on herbal medicinal products in the paediatric population: data from the PhytoVIS study

**DOI:** 10.1007/s00431-019-03532-3

**Published:** 2019-12-11

**Authors:** Karen Nieber, Esther Raskopf, Johanna Möller, Olaf Kelber, Robert Fürst, Kija Shah-Hosseini, Jaswinder Singh, Karin Kraft, Ralph Mösgens

**Affiliations:** 1 Institute of Pharmacy, University of Leipzig, Brüderstr. 34, 04103 Leipzig, Germany; 2 Kooperation Phytopharmaka GbR, Plittersdorfer Str. 218, 53173 Bonn, Germany; 3 Institute of Medical Statistics and Computational Biology, Faculty of Medicine, University of Cologne, Kerpener Str. 62, 50937 Cologne, Germany; 4 ClinNovis GmbH, Genter Str. 7, 50672 Cologne, Germany; 5 Bayer Consumer Health, Research & Development, Phytomedicines Supply and Development Center, Steigerwald Arzneimittelwerk GmbH, Havelstr. 5, 64295 Darmstadt, Germany; 6 Institute of Pharmaceutical Biology, Goethe University Frankfurt, Max-von-Laue-Str. 9, 60438 Frankfurt, Germany; 7 Chair of Naturopathy, University Medicine Rostock, Ernst-Heydemann Str. 6, 18057 Rostock, Germany

**Keywords:** Herbal medicinal products, Pharmaco-epidemiological study, Children, Adolescents, PhytoVIS project

## Abstract

In paediatrics, clinical study data are limited, especially on herbal medicinal products. To address this gap, 2063 datasets from the paediatric population were evaluated in the PhytoVIS data base. By screening for paediatric data, information on indication, gender, treatment, co-medication and tolerability were evaluated. The majority of patients was treated because of common cold, fever, digestive complaints, skin diseases, sleep disturbances and anxiety. The perceived effect of the therapy was rated in 84% of the patients as very good or good without adverse events. The data shed light on a still neglected field of phyto-pharmacotherapy by giving information on the use of herbal medicines in an unselected cohort of paediatric patients. The results confirm the good clinical effects and safety of herbal medicinal products in this patient population and show that they are widely used in Germany.

**Table:** 

**What is Known:** • In Germany, about 85% of children receive one or more herbal medicinal products per year. • Despite international initiatives to promote clinical research in paediatrics, there are still many gaps of knowledge in the use of drugs in paediatrics.	
**What is New:** • The PhytoVIS project evaluated 2063 data sets from the paediatric population using herbal medicinal products. • The majority of patients was treated because of common cold, fever, digestive complaints, skin diseases, sleep disturbances and anxiety, and 84% of the patients rated the therapy as very good or good without adverse events.

**What is Known:**

• In Germany, about 85% of children receive one or more herbal medicinal products per year.

• Despite international initiatives to promote clinical research in paediatrics, there are still many gaps of knowledge in the use of drugs in paediatrics.

**What is New:**

• The PhytoVIS project evaluated 2063 data sets from the paediatric population using herbal medicinal products.

• The majority of patients was treated because of common cold, fever, digestive complaints, skin diseases, sleep disturbances and anxiety, and 84% of the patients rated the therapy as very good or good without adverse events.

## Introduction

More and more parents are considering the use of herbal medicinal products (HMPs) to maintain the health of their children and to treat their diseases [1]. In Germany, which has one of the longest traditions of HMPs as registered medicinal products worldwide, about 85% of children receive at least one or more HMP(s) per year [2]. Parents seek advice from paediatricians and other primary care physicians or pharmacists on the safety and therapeutic effect of herbal medicines for children. However, evidence-based advice is often difficult because clinical studies on HMPs in children frequently leave open questions about efficacy, safety and side effects [3].

Although some of the safety information can be provided by randomized controlled trials (RCTs), they are often limited in terms of sample size and in length of follow-up [4–7]*.* This implicates that safety can best be answered by pharmaco-epidemiological studies [8, 9] or by individual case safety reports [10]. This is particularly relevant for children among whom the use of drugs is frequently off-label but recorded in routine care [11]. Although the field of pharmaco-epidemiology has grown substantially in the last 20 years, very few researchers focus on the paediatric patient group and on HMPs, where data on clinical studies are rare [12].The PhytoVIS database was created as a tool by which the experiences of patients who acquired HMPs in pharmacies could be recorded. In this review, the data were evaluated with respect to the paediatric population.

## Methods

The data were collected by means of a retrospective, anonymous, one-off survey consisting of 20 questions on the user’s experience with HMPs. The questions included complaints/disease, information on drug use, concomitant factors/diseases as well as basic patient data.

Trained interviewers performed the interviews in pharmacies and doctor’s offices. Data were collected in the Western Part of Germany between April 2014 and December 2016. The only inclusion criterion was the intake of herbal drugs in the last 8 weeks before the individual interview. The primary endpoint was the effect and tolerability of the products according to the user (determined by the CGI-E, Clinical Global Impression Scale-Efficacy) [13]. Secondary endpoints were source of supply and recommendation of the products. Data were collected by the Institute of Medical Statistics, Informatics and Epidemiology, Cologne, Germany (now: Institute of Medical Statistics and Computational Biology, IMSB) using secuTrial®, a professional, fully browser-based tool for collecting patient data in clinical or non-interventional studies and patient registries. The patient data collected did not allow drawing conclusions about the interviewed person. To further enhance anonymity, the age was clustered for evaluation. For paediatric patients, this was done according to the European Medicines Agency guideline CPMP/ICH/2711/99. Statistical analysis was done descriptively, with frequency and percentages or mean + SD (if applicable) using the statistical software IBM SPSS Statistics for Windows (version 22.0, IBM Corp., Armonk, NY, USA). Demographic and other baseline characteristics (sex, age, concomitant disease, dispensing site and recommendation) as well as all therapeutical effects and safety variables were analysed using descriptive statistics. Categorical data were expressed as absolute or percentage of frequency. Missing values were not carried forward but regarded as missing values. As this was a one-off online survey on past application experience, there were no statutory notification obligations under the German Medicinal Products Act (Arzneimittelgesetz) and the Act on Medical Devices (Medizinproduktegesetz). With a positive answer to the introductory question, the patient gave oral consent to participate in the survey.

The PhytoVIS database, consisting of 20,870 patients in total, was screened for data on paediatric patients. The questionnaires were evaluated regarding epidemiological data on gender, age and indication as well as on the perception of the used HMPs after elimination of non-herbal products and formation of indication groups. Excel 2010 software was used for preparation of the figures.

## Results

**Fig. 1:**
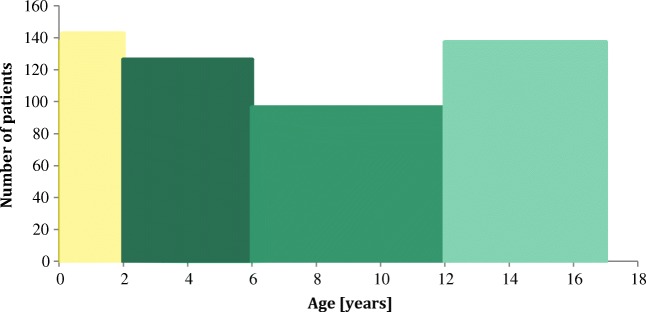
Age distribution of 2063 datasets from the paediatric population

**Fig. 2:**
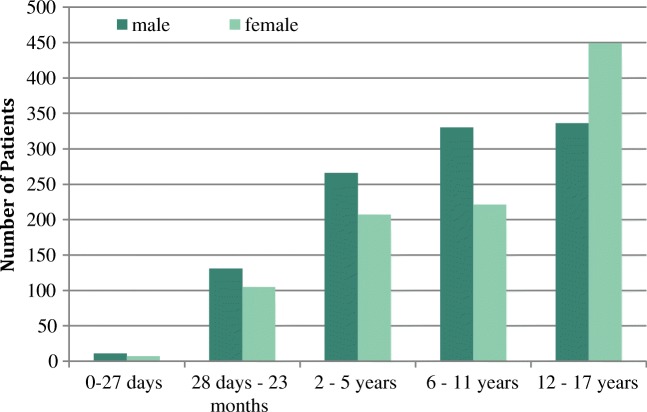
Gender-dependent distribution of 2063 datasets from the paediatric population

Overall, 2063 datasets from the paediatric population were evaluated, thereof 254 from patients below 2 years (12.3%), 473 from patients aged 2–5 years (22.9%), 551 from age 6–11 years (26.7%) and 785 from age 12–17 years (38.1%). A conversion of the age cluster in patients per year of age showed a slightly different allocation (Fig. 1). The evaluation of the gender frequency revealed an almost equal distribution (male, 52% vs. female 48%). Interestingly, in the lower age groups (0 to 11 years), more male patients were treated with herbal medicines, whereas among the adolescents (12–17 years), females clearly outweighed them (Fig. 2).

### Indications

**Fig. 3:**
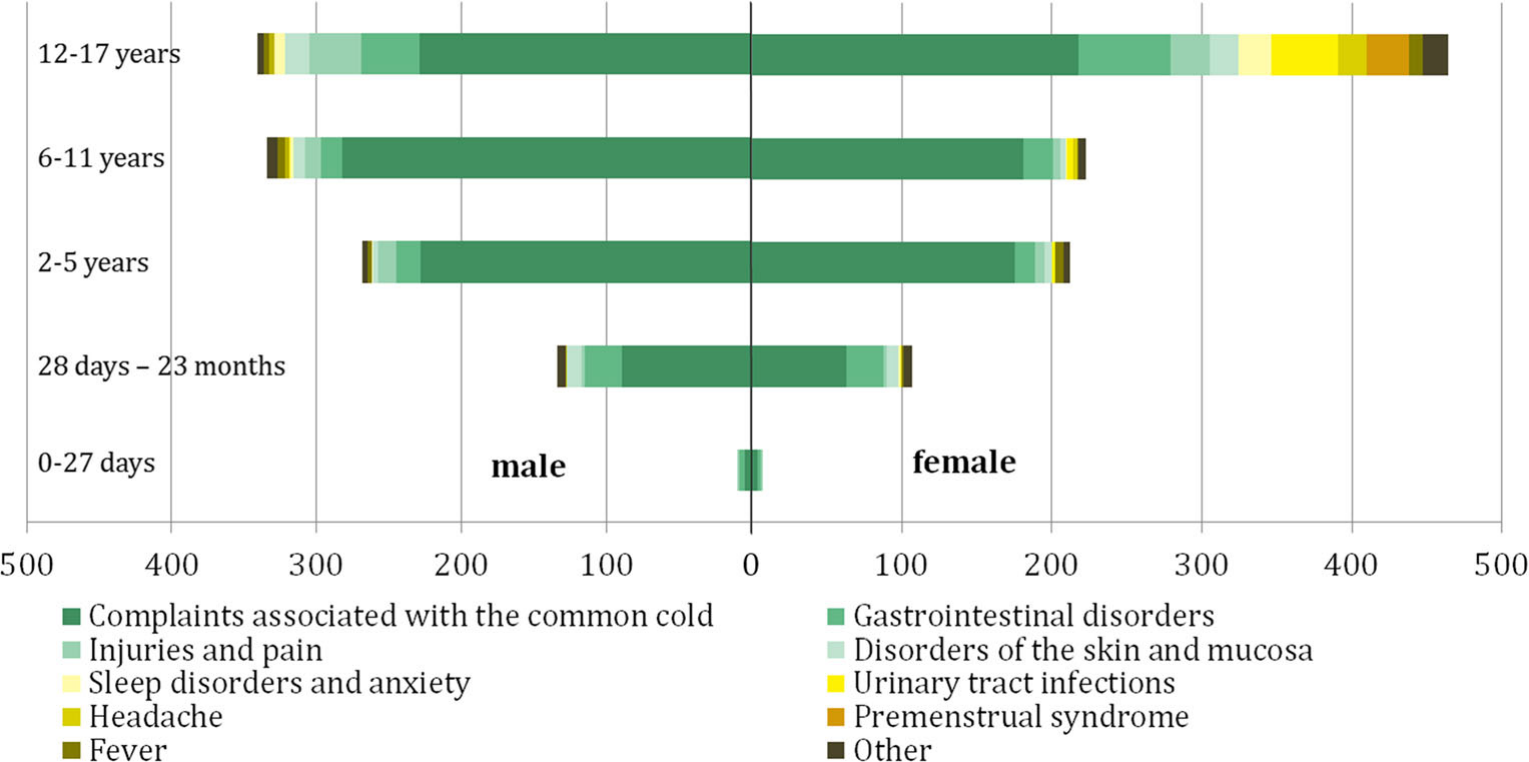
Indications depending on age of 2063 data sets from the paediatric population

The majority of patients (67.7%) were treated because of common cold and fever, 13.6% due to digestive complaints, 4.9% because of skin diseases, 3.5% due to sleep disturbances and anxiety, and 10.3% because of other complaints. Interestingly, the intake of HMPs increased with age and showed a maximum in the adolescents (Fig. 3). A co-medication was documented in 24.9% of the patients.

### Perceived effects depending on indication

**Fig. 4:**
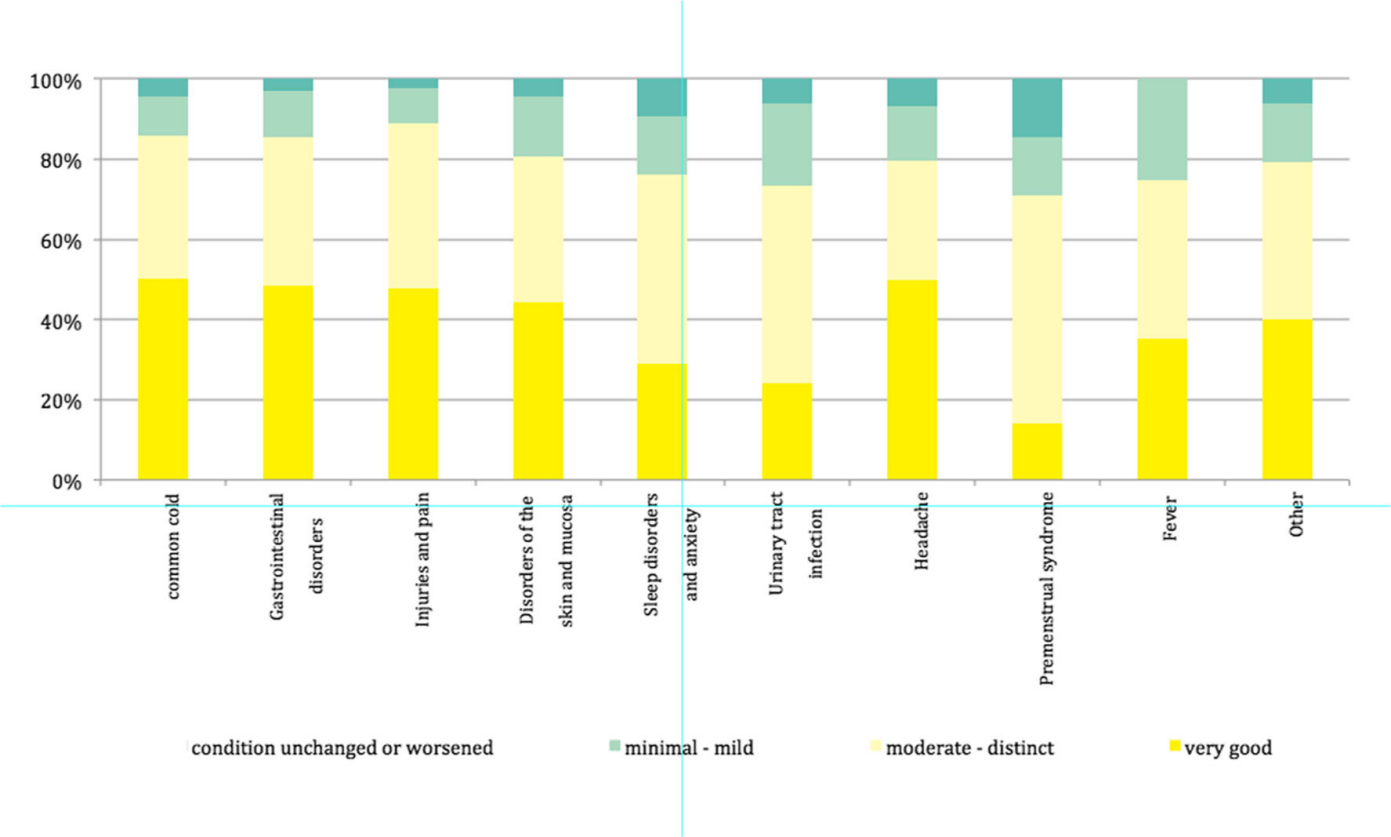
Perceived effects depending on indication of 2063 evaluated datasets from the paediatric population

The perceived effect of the therapy was rated as very good in 48.4% of the patients, good to moderate in 36.8%, modest in 10.8% and missing in 4.0%. It is noteworthy that the number of respondents who assessed the effect as very good or moderate did not differ with respect to the indications (Fig. 4).

### Adverse reactions depending on age

Out of all patients, 93.7% experienced no adverse events. Only 0.8% of all patients reported a marked impairment due to side effects. The tolerability did not seem to differ between the age groups.

## Discussion

The age- and gender-specific evaluation of the data from the PhytoVIS project provides insight into a hitherto scarcely investigated field of pharmacotherapy and gives a picture of the use of HMPs in an unselected cohort of paediatric patients.

The lack of availability of appropriate medicines for children is an extensive and well-known problem [13, 14]. Paediatricians and other physicians who take care of the paediatric population are primarily and very frequently exposed to cope with this problem: in more than half of the children, off-label or unlicensed medicines are prescribed [15, 16].

Phytotherapy is suitable for healing, relief and prevention of mild to moderate diseases and often provides a well-tolerated therapy with a very low number of side effects, especially for children and adolescents. If used correctly, HMPs are excellently tolerated. Depending on the grade of the disease, the indication-specific HMPs are used either alone or combined with chemically synthesized medicines. In addition to HMPs, non-drug related procedures play an important role, especially in children. Thus, the treatment of headache can be successful with non-pharmacological techniques, i.e. transcendental meditation, hypnotherapy and progressive muscle relaxation [17]. Further methods such as clown therapy also show surprisingly good effects in children, especially in anxiety [18].

Safe and effective pharmacotherapy in paediatric patients requires the timely development of information on the proper use of medicinal products in children of various ages. As maturation plays a crucial role in the pharmacology of a drug, it is important to check whether the risk estimates between the drug exposure and the adverse drug reaction fluctuates by age. In paediatric drug research, therefore, it is recommended not only to study the effect of a drug in the paediatric population as a whole (0–18 years), but also to repeat the analysis within different age categories. The present study did not reveal any age-dependent risks of the HMPs used.

## Strengths and weaknesses

The strength of this study is the systematic assessment of pharmaco-epidemiological data in children, with a broad inclusion criterion. However, we only disposed about the subjective assessment by the interviewed patients, but no objective data on clinical examination and no observation of clinical effect by the treating physicians were available. Despite the advanced data collection techniques used, which should decrease the frequency of systematic and random measurement errors, questionnaires are still subject to bias as a result of self-reporting. Also, the formation of indication groups was necessary due to numerous and heterogeneous outcomes. The interviews were performed only in the Western, but not in the Eastern part of Germany. Therefore, the results cannot be generalized, neither to all Germany nor to other European countries.

## Conclusion

The evaluation of the 2063 datasets from the paediatric population provides a better understanding of the use of HMPs in this age population. It shows therapeutic usefulness of HMPs in a prescription as well as in a self-medication setting. Nevertheless, further well-designed trials are required to build up an adequate evidence base and to provide accurate information on dosing in order to assist clinical decision-making and to ensure the safe use of HMPs for children.

## Abbreviations

CGI-E: Clinical Global Impression Scale-Efficacy
HMPs: Herbal medicinal products
IMSB: Institute of Medical Statistics and Computational Biology
MedDRA: Highly specific standardized medical terminology
RCTs: Randomized controlled trials
